# Binding Affinity and Capacity for the Uremic Toxin Indoxyl Sulfate

**DOI:** 10.3390/toxins6020416

**Published:** 2014-01-24

**Authors:** Eric Devine, Detlef H. Krieter, Marieke Rüth, Joachim Jankovski, Horst-Dieter Lemke

**Affiliations:** 1 eXcorLab GmbH, Industrie Center Obernburg, Obernburg 63784, Germany; E-Mails: devineer@gmail.com (E.D.); marieke.rueth@excorlab.de (M.R.); 2 University Hospital Würzburg, Department of Medicine, Division of Nephrology, Würzburg 97080, Germany; E-Mail: krieter_d@medizin.uni-wuerzburg.de; 3 Medizinische Klinik IV, Campus Benjamin Franklin, Charité—Universitätsmedizin Berlin, Berlin 10117, Germany; E-Mail: joachim.jankowski@charite.de

**Keywords:** kidney disease, uremic toxin, protein binding, ionic strength, hemodialysis

## Abstract

Protein binding prevents uremic toxins from removal by conventional extracorporeal therapies leading to accumulation in maintenance dialysis patients. Weakening of the protein binding may enhance the dialytic elimination of these toxins. In ultrafiltration and equilibrium dialysis experiments, different measures to modify the plasma binding affinity and capacity were tested: (i), increasing the sodium chloride (NaCl) concentration to achieve a higher ionic strength; (ii), increasing the temperature; and (iii), dilution. The effects on the dissociation constant *K*_D_ and the protein bound fraction of the prototypical uremic toxin indoxyl sulfate (IS) in plasma of healthy and uremic individuals were studied. Binding of IS corresponded to one site binding in normal plasma. *K_D_* increased linearly with the NaCl concentration between 0.15 (*K*_D_ = 13.2 ± 3.7 µM) and 0.75 M (*K*_D_ = 56.2 ± 2.0 µM). Plasma dilution further reduced the protein bound toxin fraction by lowering the protein binding capacity of the plasma. Higher temperatures also decreased the protein bound fraction of IS in human plasma. Increasing the NaCl concentration was effective to weaken the binding of IS also in uremic plasma: the protein bound fraction decreased from 89% ± 3% to 81% ± 3% at 0.15 and 0.75 M NaCl, respectively. Dilution and increasing the ionic strength and temperature enhance the free fraction of IS allowing better removal of the substance during dialysis. Applied during clinical dialysis, this may have beneficial effects on the long-term outcome of maintenance dialysis patients.

## 1. Introduction

The uremic syndrome is attributed to the accumulation of a large number of compounds, which in healthy individuals are excreted by the kidney. These compounds are called uremic retention solutes or uremic toxins since they have deleterious effects on the human organism. Indoxyl sulfate (IS, 213 Da) is a prototypical protein-bound uremic toxin [1]. In chronic kidney disease, particularly in patients on maintenance dialysis, IS is associated with cardiovascular outcome and mortality [2,3,4]. 

Albumin (molecular weight 66.5 kDa) is the most abundant plasma protein with a concentration of about 570 µM (38 g/L) [5,6]. It is a carrier protein for many hydrophobic compounds in plasma [6,7]. Numerous uremic toxins bind specifically to the Sudlow’s sites I and II of human serum albumin [8], thereby, leading to impaired binding of drugs [8,9]. IS binds to the Sudlow’s site II in subdomain IIIA with an association constant *K*_A_ = 9.1 × 10^5^ to 16.1 × 10^5^ M^−1^ (corresponding to a dissociation constant *K*_D_ = 0.6 to 1.1 µM) [10,11]. In principle, the protein binding of uremic toxins is reversible since it is mainly driven by electrostatic and/or van der Waals forces [9,12]. Due to its high protein bound fraction and high distribution volume resulting in low dialytic clearance [13,14,15,16,17,18,19,20], IS is poorly removed by existing extracorporeal renal replacement therapies, such as hemodialysis. 

Several studies have investigated the protein binding of uremic toxins exclusively in albumin solution as a surrogate for plasma [10,11,21,22]. Such an approach does not take into account a possible competition of different plasma compounds for the limited number of binding sites. The present study was performed to quantitatively describe the binding of IS in more physiological conditions as in [23], and to apply different experimental settings in order to develop the basis for methods which enhance the clearance of protein bound substances during clinical hemodialysis. 

## 2. Results

### 2.1. Binding of IS to Normal Human Plasma at Different Temperature and Ionic Strength

A rise in temperature from 25 °C to 37 °C significantly reduced the fraction of protein bound IS in both 0.15 M NaCl (90% ± 2% to 86% ± 1%, *p* < 0.05) and 0.61 M NaCl solution (83% ± 2% to 77% ± 2%, *p* < 0.01).

**Figure 1:**
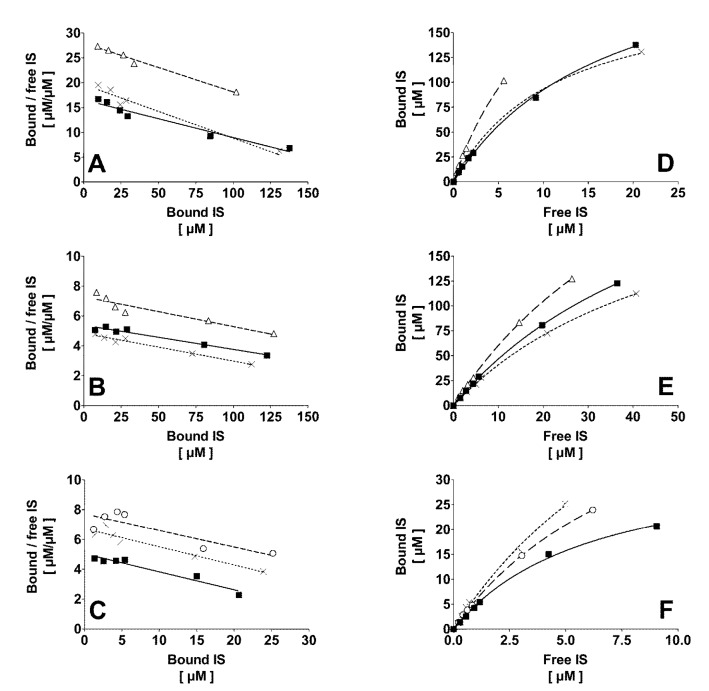
Scatchard plot of the protein binding of indoxyl sulfate (**A**–**C**) and corresponding binding curves (**D**–**F**) in normal human plasma. Plasma was 1:2 diluted and incubated at 0.15 M NaCl (**A**,**D**) and 0.75 M NaCl (**B**,**E**). 1:10-diluted plasma was incubated only at 0.15 M NaCl (**C**,**F**). Each experiment was performed with plasma from three different donors (squares: donor 1, triangles: donor 2, crosses: donor 3, and circles: donor 4). Indoxyl sulfate bound to one high affinity binding site in normal plasma. Regression coefficients *r*^2^ were: (**A**) 0.95, 0.98, and 0.96; (**B**) 0.96, 0.89, and 0.97 for donors 1, 2, and 3, respectively; (**C**) 0.94, 0.92 and 0.75 for donors 1, 3 and 4, respectively. Regression coefficient *r^2^* of the binding curves; (**D**–**F**) was always 1.00.

**Figure 2:**
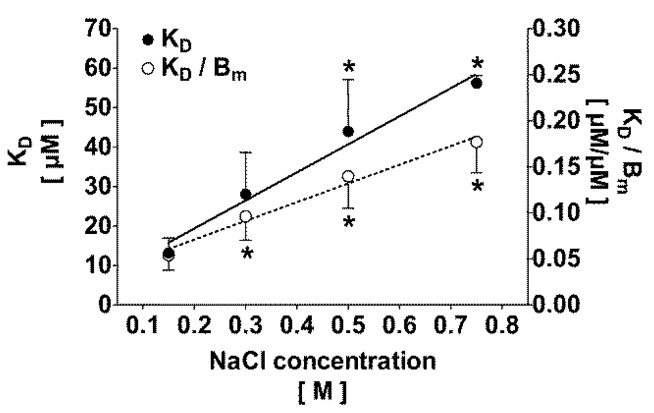
Effect of the ionic strength on the dissociation constant *K*_D_ (solid line) and the ratio *K*_D_*/B*_m_ (dotted line) for indoxyl sulfate in 1:2-diluted plasma. *K*_D_ and *K*_D_*/B*_m_ correlate with the ionic strength according to *K*_D_ = 71.2 × NaCl concentration + 5.1 and *K*_D_/*B*_m_ = 0.20 × NaCl concentration + 0.03, respectively. *p < 0.05 *versus* 0.15 M NaCl.

Mathematical modeling (Equation (2) *versus* Equation (3)) revealed that, independently of the ionic strength, the binding of IS in normal human plasma best fits to a one binding site model. The binding constants of a second binding site in plasma were calculated to be irrelevant. One site-specific binding curve (Equation (2)) at 0.15 M and 0.75 M NaCl are presented in Figure 1D,E, respectively. Scatchard plots of IS in normal human plasma are shown for 0.15 M and 0.75 M NaCl (Figure 1A,B, respectively). Similar results were obtained for 0.30 M and 0.50 M NaCl (data not shown). Binding constants calculated from the Scatchard plot (*K*_D_ = 10.9 ± 2.0, 22.1 ± 6.5, 39.7 ± 11.1 and 54.9 ± 5.9 µM; *B*_m_ = 228 ± 52, 251 ± 33, 296 ± 54, and 318 ± 54 µM, at 0.15 M, 0.30 M, 0.50 M, and 0.75 M, respectively) did not significantly differ from the values obtained from non-linear regression using Equation (2). As shown in Figure 2, an increase of the ionic strength highly correlated with *K*_D_ (*r*² = 0.98; *p* < 0.05) and the ratio *K*_D_*/B*_m_ (*r*² = 0.98; *p* < 0.01). The maximal binding capacity *B*_m_ slightly increased with the ionic strength. However, compared to isotonic NaCl concentration, this finding was statistically significant only at the highest ionic strength of 0.75 M. Increasing the ionic strength resulted in a significant decrease (*p* < 0.05) of the protein bound fraction from 95% ± 1% at 0.15 M NaCl to 91% ± 2%, 87% ± 3%, and 84% ± 2% at 0.30 M, 0.50 M, and 0.75 M NaCl, respectively, at low toxin-albumin ratio (α = 0.1). The theoretical protein bound fraction for α = 0.1 was 94% ± 2%, 90% ± 2%, 87% ± 3%, and 84% ± 3% at 0.15 M, 0.30 M, 0.50 M, and 0.75 M, respectively. Experimental and theoretical protein bound fractions highly correlated (*r*² = 0.99; *p* < 0.0001). 

### 2.2. Correlation of the Ionic Strength with the Protein Bound Fraction of IS in Uremic Plasma

**Figure 3:**
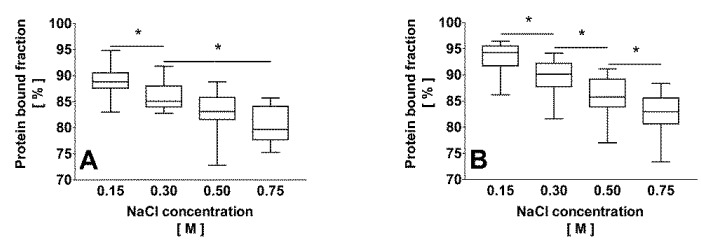
Effect of an increased NaCl concentration on the protein bound fraction of indoxyl sulfate in uremic (**A**); *n* = 15, free and bound native toxin concentrations) and healthy (**B**); *n* = 18 (3 different donors spiked with 6 different toxin concentrations), free and bound toxin concentrations) human plasma. Increasing ionic strength led to lower protein bound fractions of indoxyl sulfate in both uremic and normal plasma. * *p* < 0.05.

With increasing ionic strength, the protein bound fraction of IS decreased significantly in plasma of uremic and healthy individuals (Figure 3A,B, respectively). Compared with uremic plasma, the protein bound fraction in normal plasma was about 4% higher at 0.15 M and 0.30 M NaCl (*p* < 0.01). There was no significant difference between uremic and IS added healthy plasma regarding the mean total IS concentration (69.4 ± 35.5 µM *versus* 56.7 ± 54.5 µM, respectively). Considering Equation (1) with an experimental albumin concentration of 286 µM after 1:2 dilution, i.e., hypothesizing *B*_m_ being constant at 286 µM, a higher NaCl concentration in uremic plasma correlated with *K*_D_ for IS (27.3 ± 8.6 µM, 37.1 ± 10.6 µM, 46.5 ± 16.9 µM, and 55.9 ± 15.3 µM at 0.15 M, 0.30 M, 0.50 M, and 0.75 M NaCl, respectively (*r*² = 0.40; *p* < 0.0001)).

### 2.3. Effect of Dilution and Higher Ionic Strength on Toxin Binding in Human Plasma

For a given NaCl concentration, 1:2 and 1:10 dilution did not affect the binding affinity *K*_D_ (see Table 1), while the maximal binding capacity *B*_m_ was decreased significantly (*p* < 0.05), being equivalent to the albumin concentration (238 ± 90 µM for 1:2 and 48 ± 18 µM for 1:10 dilution). Fitting models (Equation (2) *versus* Equation (3)) confirmed a single high affinity binding site in 1:10 diluted human plasma, as also shown by the corresponding Scatchard plot (Figure 1C). Appropriate results were obtained for 0.50 M NaCl (data not shown). Binding constants calculated by Scatchard plot (*K*_D_ = 8.5 ± 0.4 and 48.5 ± 6.7 µM; *B*_m_ = 55 ± 14 and 124 ± 38 µM, at NaCl 0.15 M and 0.50 M, respectively) did not differ from the values obtained by non-linear regression using Equation (2). Experimental and theoretical protein bound fractions highly correlated (*r*² = 0.98; *p* < 0.0001).

**Table 1:** Binding constants *K_D_* and *B_m_* and protein bound fraction of indoxyl sulfate in 1:2 and 1:10-diluted normal human plasma. Abbreviations: [NaCl], sodium chloride concentration; α, toxin-protein ratio. Values are mean ± SD (*n* = 3). Plasma dilution modified only *B*_m_ and, thus, the ratio *K*_D_*/B*_m_. The effects of higher [NaCl] on the protein bound fractions of IS, as assessed by Equation (1), are also shown. ^(a)^ *p* < 0.05 *versus* 1:2 dilution; ^(b)^ *p* < 0.05 *versus* 0.15 M NaCl; ^(c)^ *p* < 0.05 *versus* theoretical bound fraction.

[NaCl] [M]	Dilution factor	KD [µM]	Bm [µM]	KD/Bm	Theoretically bound fraction α = 0.1 [%]	Experimentally bound fraction α = 0.1 [%]
0.15	1:2	13.4 ± 3.6	261 ± 64	0.05 ± 0.02	94 ± 2	95 ± 2 ^(c)^
0.50	1:2	40.1 ± 18.4	297 ± 64	0.13 ± 0.05 ^(b)^	87 ± 4 ^(b)^	88 ± 4 ^(b)^
0.15	1:10	8.9 ± 2.7	59 ± 24 ^(a)^	0.16 ± 0.02 ^(a)^	85 ± 1 ^(a)^	86 ± 3 ^(a)^
0.50	1:10	44.7 ± 18.3	109 ± 27 ^(a)^	0.41 ± 0.11 ^(a,b)^	70 ± 5 ^(a,b)^	73 ± 6 ^(a,b)^

## 3. Discussion

IS is a prototypical, highly protein bound uremic toxin associated with the morbidity and mortality of maintenance dialysis patients [2,3,4]. *In vitro* experiments have shown its implication in bone and cardiovascular disorders frequently observed in uremic patients. Bone disease is elicited by IS by inhibiting osteoclast differentiation and function, and by inducing osteoblast resistance to parathyroid hormone [24,25]. Cardiovascular disease is promoted by IS induced oxidative stress, leading to inhibition of endothelial cell proliferation and wound repair [26,27]. Although it is a rather small compound (213 Da), IS removal by hemodialysis is impaired due to the binding to larger proteins, which, in contrast to the free toxin, are prevented from passing dialysis membranes. Therefore, releasing IS from its protein binding sites would promise more efficient removal, but would require a modification of existent dialysis procedures. To achieve such a goal, investigating the effects of clinically applicable methods on protein binding represents an obvious initial approach. 

As demonstrated by equilibrium dialysis in aqueous solution, IS binds to albumin with an affinity *K*_D_ of 0.6 to 1.1 µM [10,11]. The present experiments exploring ultrafiltration, which is closer to clinical dialysis, demonstrated that in human plasma at physiological ionic strength and room temperature IS is bound to proteins with a higher *K*_D_ of 13.4 ± 3.6 µM. This much lower affinity for IS in plasma may be best explained by the presence of potential competing ligands, such as fatty acids, which may alter the binding properties of albumin by occupying the binding site [28]. A methodological artifact can be excluded because comparable protein bound fractions of IS were obtained with both ultrafiltration and equilibrium dialysis. Together with protein adsorption onto the semipermeable dialysis membrane [29], ultrafiltration of plasma water leads to an increase of the protein concentration in the retentate and, thus, a shift in the equilibrium. This phenomenon is well known from clinical hemodialysis, especially during post-dilution hemodiafiltration, a dialysis procedure, which combines excess ultrafiltration of plasma water to diffusion, leading to protein concentration within the dialyzer near the membrane surface. The high variability of *K*_D_ observed in the present experiments (Figure 2) reflects the interindividually different concentrations of competing ligands, which alter the ability of plasma from different individuals to bind toxins [30,31,32].

In the present experiments a single class of high affinity binding sites were found for IS in plasma. This finding was supported by comparing one site and two site specific binding models (Equations (2) and (3), respectively). Moreover, no difference was found between the Scatchard plot (i.e., interception with X-axis) and non-linear regression (Equation (2)) when calculating *B*_m_. In both cases, *B*_m_ was in the range of the varying albumin concentrations. As calculation of the ratio of bound and free ligand from the Scatchard plot may promote errors in the resulting binding constants, the results obtained by non-linear regression were preferred and further reinforced by the information provided from the Scatchard plot. In previous publications it was found that IS binds to two binding sites in human serum albumin solution (one with high, the other with low affinity) [23,33]. The difference in the number of binding sites between plasma and albumin solution according to the results of the present study and the literature, respectively, may be best explained by the presence of multiple competitors, such as fatty acids, which are absent in purified albumin solution and occupy the low affinity binding site in plasma [32,34]. As previous studies have shown that IS binds to Sudlow’s site II of albumin [10,11], it must be bound to the same binding site also in plasma.

Increasing the NaCl concentration from physiological 0.15 M to 0.75 M effectively decreased the protein bound fraction of IS in both normal plasma (from 93% ± 3% to 83% ± 4%) and plasma from uremic patients (from 89% ± 3% to 81% ± 3%). The lower protein bound fraction in uremic plasma can be explained by competition of other uremic toxins, such as *p*-cresyl sulfate [19], which are not present in healthy human plasma, for the albumin-binding site. Supporting this argument, compared to IS, the native *p*-cresyl sulfate concentration in uremic plasma (90 ± 28 µM, data not shown) and its protein bound fraction (91% ± 2% at 0.15 M NaCl, data not shown) were in the same range. The decrease of the protein bound fraction of IS was associated with a linear increase of *K*_D_ being 56.2 ± 2.0 µM at 0.75 M NaCl *versus* 13.4 ± 3.6 µM at 0.15 M. An increase of the ionic strength may result in a modification of electrostatic charges within the binding pocket of albumin and/or a modification of the protein conformation interfering with IS binding. As no decrease of *B*_m_ at the given NaCl concentrations was observed, it can be concluded that electrostatic interactions definitely are involved. This result is in agreement with previous findings, which demonstrated that increasing the ionic strength to 0.30 M NaCl decreased the binding affinity of serum albumin for both *p*-cresol and *p*-cresyl sulfate [26]. Furthermore, the same was reported for naproxen [35], a drug known to bind to Sudlow’s site II [36].

**Figure 4:**
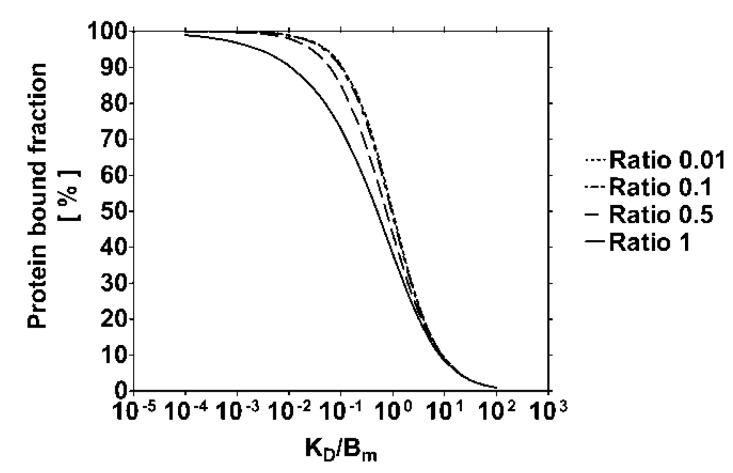
Theoretical course of the protein bound fraction as a function of *K*_D_/*B*_m_ for different toxin-protein ratios α according to Equation (1). α and *K*_D_*/B*_m_ were varied from 0.01 to 1 mol/mol and from 10^−4^ to 10^2^ mol/mol, respectively. The binding curve corresponds to a single high-affinity binding site on protein.

Two to tenfold dilution of plasma also showed to be effective for decreasing the protein bound fraction of IS. This effect was additive to that of an increased NaCl concentration and can be explained by an increase of *K*_D_ with rising ionic strength and a decrease of *B*_m_ with dilution. Both effects cumulate and lead to an increase in the ratio *K*_D_*/B*_m_ and, *a fortiori,* to a reduction of the protein bound fraction (Figure 4). Clinical and experimental observations support this phenomenon [11,37,38]. Low serum albumin concentrations in uremic patients were found to correlate with a higher free fraction of *p*-cresyl sulfate [29]. The same authors confirmed this finding by testing different bovine serum albumin concentrations *in vitro* [37], supporting the data of the present study, which demonstrated that plasma dilution did not influence *K*_D_. Weisiger *et al.* reported that the apparent affinity of human serum albumin for bilirubin was inversely proportional to the protein concentration, although, the affinity was rather constant for albumin concentrations greater than 100 µM [38]. The binding affinity of human serum albumin for dansylsarcosine, a marker ligand of albumin site II, was similar when using 40 µM or 200 µM albumin [11]. Thus, it is important to take the protein concentration or plasma dilution into consideration when comparing the protein bound fractions from different experiments or clinical studies. Therefore, in the present binding inhibition experiments with IS and NaCl in normal and uremic plasma, the same plasma dilution always was applied (native plasma was diluted 1:2). 

Increasing the temperature from room to body temperature further decreased the protein bound fraction of IS in normal human plasma. Thus, our data are in agreement with [23]. Confirming Bergé-Lefranc *et al.*, who reported that enhancing the temperature reduced the affinity of human serum albumin for both *p*-cresol and *p*-cresyl sulfate without changing the protein structure in this temperature range [39], this finding is in accordance with the Van’t Hoff equation. Thus, compared to body temperature, performing the present experiments at room temperature should have overestimated the protein bound fraction and the binding affinity of IS in human plasma. Therefore, the influence of increased ionic strength during clinical dialysis (body temperature) should be greater than observed in the experiments. Based on the removal of only the free toxin fraction during hemodialysis [21,22], an increase of the free fraction of IS in uremic plasma from 11% (0.15 M NaCl) to 19% (0.75 M NaCl) should double its plasma clearance. Different modifications of the setup are conceivable, which theoretically allow the application of higher ionic strengths in plasma or blood during clinically practiced hemodialysis. 

Finally, it was verified for normal plasma if Equation (1) is suited to allow an accurate prediction of the experimentally bound fraction of IS from the binding constants independently of the plasma dilution. At low toxin concentrations, the protein bound fraction correlated directly with the binding affinity *K*_D_ and the maximal binding capacity *B*_m_ and, thus, with the protein concentration [40]. Advantageously reflected by Equation (1), the binding affinity in uremic plasma can be assessed when knowing the protein bound fraction, the toxin and the receptor protein concentrations, which all can be determined experimentally. As albumin was not measured in the plasma samples, a mean albumin concentration of 570 µM, which is in accordance with previous studies in dialysis patients [5,19,41], and a *B*_m_ equal to the human serum albumin concentration were taken as assumptions for the calculations. Equation (1) yielded that increasing the NaCl concentration in uremic plasma from 0.15 M to 0.75 M NaCl enhanced *K_D_* for IS from 27.3 ± 8.6 µM to 55.9 ± 15.3 µM. This estimated binding affinity reflects the apparent affinity of uremic plasma for IS and is similar to that found in normal plasma. A methodological artifact was excluded by verifying the stability of albumin during freezing of the plasma samples. In three plasma samples, a constant human albumin concentration was confirmed before freezing at −20 °C and after thawing (43 ± 6 g/L, *p* = 0.18).

To note, performing direct binding studies in native uremic plasma is difficult to interpret because of the competition between the multiple protein bound uremic toxins present in plasma and any newly added ligand [8,9,10,11,30]. Alternatively, free and bound toxin concentrations, and the binding constants in plasma from a large number of uremic patients, could be assessed. Drawback of this approach is the huge array of required samples and the interindividual variability interfering with accurate calculations. 

Some shortcomings of the present study need to be addressed. The experiments may have been confounded by some differences in anticoagulation, which were due to a matter of blood availability. Despite the fact that De Smet *et al.* reported interference of heparin with free *p*-cresol determination [42], an effect of anticoagulation on the protein binding in the present setting must be doubted as the blood was heparinized during donation. Thus, equilibrium of fatty acids binding should have been already reached upon experimentation. Furthermore, to keep the plasma dilution constant between experiments, the amount of PBS with physiological pH was adapted. As plasma is a natural buffer solution, this method should not have had a major impact on the pH stability of the samples. Finally, different to uremic plasma, normal plasma needed to be spiked with different amounts of IS available in form of a potassium salt to verify the effect of increased ionic strength *in vitro*. With respect to the NaCl concentration (at least 150 mM), the increase in ionic strength resulting from the introduced potassium (the highest concentration being about 0.15 mM) was negligible.

In summary, uremic and normal plasma do not differ in terms of the binding affinity for protein bound toxins with respect to increased ionic strength as demonstrated using the example of the prototypical IS. Furthermore, Equation (1) is a useful tool to describe binding properties of similar toxins in normal and uremic plasma.

## 4. Experimental Section

### 4.1. Modeling of the Protein Bound Toxin Fraction

Models characterizing the behavior of protein bound solutes during dialysis have been developed based on the removal of the unbound toxin fraction from bovine serum albumin solution [21,22]. As the binding of uremic toxins, such as IS, follows the law of mass action, it is possible to describe their protein bound fraction as a function of the binding constants (*K*_D_, and *B*_m_*,* the maximal binding capacity) and the toxin-protein ratio α = *L*_T_/*B*_m_, according to Equation (1), where *L*_T_ is the total toxin concentration (also refer to Figure 1):

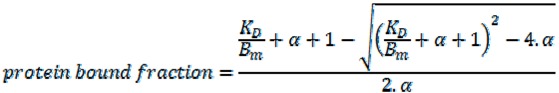
(1)
This equation, reflecting one site specific binding, was adapted [22] by isolating the ratio *K*_D_*/B*_m_. For details of the calculation refer to the Supplemental Data section, Equation (1). 

### 4.2. Binding Capacity of Human Plasma at Different Temperature and Ionic Strength

Experiments using plasma obtained from four different donors by centrifugation of whole blood were performed at two different ionic strengths. Firstly, 17.5 mL freshly donated heparinized (5 IU/mL) plasma from a single healthy donor was diluted with the same volume of phosphate buffered saline pH 7.4 (PBS pH 7.4; Sigma Life Science, Sigma-Aldrich, Steinheim, Germany). Secondly, to obtain euvolemic plasma with higher ionic strength, 17.5 mL heparinized plasma was mixed with 8.75 mL PBS pH 7.4 and 8.75 mL 2.0 M NaCl (sodium chloride for analysis, Merck, Darmstadt, Germany), resulting in 1:2 diluted plasma of 0.61 M NaCl concentration. Both preparations were dialyzed (flow rate 2.1 mL/min) at 25 °C and 37 °C against a solution of 150 µM IS (indoxyl sulfate potassium salt, Sigma-Aldrich) of corresponding ionic strength (0.15 M and 0.61 M NaCl) using a mini-dialyzer with a low-flux dialysis membrane (Cuprophan^®^, cut-off 6 kDa, surface area 125 cm², inner and outer compartment each 4.6 mL; Membrana GmbH, Wuppertal, Germany). Samples for the measurement of the total toxin concentration by reversed-phase high performance liquid chromatography (RP-HPLC) were collected from the plasma and the dialysate compartments of the diaylzer when the equilibrium was reached after 1 h. The equilibrium point was determined during a prior pilot experiment (data not shown).

### 4.3. Binding Affinity of Human Plasma for IS at Different NaCl Concentrations

Experiments were performed with 0.5 mL citrate-plasma (fresh frozen plasma; Bavarian Red Cross Blood Donor Service, Munich, Germany), which was incubated for 30 min at room temperature at different NaCl concentrations in PBS pH 7.4 (NaCl concentration: 0.15 M, 0.30 M, 0.50 M, and 0.75 M). IS was added to reach a concentration of 8, 15, 23, 30, 90, and 150 µM. After adding IS and NaCl, the volume of PBS was adjusted to finally obtain 1:2 diluted plasma. The toxin/albumin ratio was kept ≤1.0 mol/mol to avoid non-specific binding. In each sample, the free and total IS concentrations were measured by RP-HPLC. Scatchard plots were generated for the free and bound IS concentrations.

The binding constants *K*_D_ and *B*_m_ were determined by nonlinear regression, comparing the one site specific binding model (Equation (2)) with the two sites specific binding model (Equation (3)) by means of the “extra sum-of-squares F test” (software GraphPad Prism, version 6.00 for Windows, GraphPad Software, La Jolla, CA, USA).

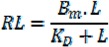
(2)
where *RL* and *L* are the bound and free toxin concentration, respectively.

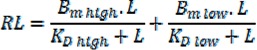
(3)
where “high” and “low” refer to the high and low affinity binding sites, respectively.

The binding constants *K*_D_ and *B*_m_ were also assessed from Scatchard plots, *i.e.*, by calculating the absolute value of the inverse slope and the interception with the X-axis, respectively.

### 4.4. Binding Capacity of Uremic Plasma for IS at Different NaCl Concentrations

After having received approval from the competent institutional review board (ethics committee of the University Hospital Würzburg, code AZ 64/12), written informed consent was obtained from 15 end-stage renal disease patients on maintenance hemodialysis in a single dialysis center (Dialysis center, Elsenfeld, Germany). Blood (7.5 mL) was collected in EDTA-tubes from each patient before dialysis. Plasma was obtained by centrifugation (10 min at 2000*g*) and stored at −30 °C. For the binding experiments, 0.5 mL uremic plasma was incubated for 30 min at different NaCl concentration (0.15 M, 0.30 M, 0.50 M, and 0.75 M NaCl) in PBS pH 7.4 at room temperature. After adding NaCl, the volume of PBS was adjusted to attain 1:2 dilution of the plasma. For each sample, the free and total IS concentrations were measured by RP-HPLC.

### 4.5. Effect of Plasma Dilution on the Binding Capacity in Vitro

These experiments were performed in citrate-plasma (fresh frozen plasma; Bavarian Red Cross Blood Donor Service) with a final dilution of either 1:2 or 1:10. Plasma (0.5 mL or 0.1 mL) was diluted with PBS, NaCl solution and IS solution to reach a final NaCl concentration of 0.15 M and 0.50 M, respectively, and a final volume of 1.0 mL. To 1:2-diluted samples IS was added to obtain the same concentrations as described above. 1:10-diluted samples contained only one fifth of the IS concentrations targeted in 1:2 diluted plasma. The samples were incubated for 30 min at room temperature, further analyzed by RP-HPLC, and the binding constants *K*_D_ and *B*_m_ were determined as previously described. 

### 4.6. RP-HPLC Method

To determine the total IS concentration, all samples were diluted 1:5 with PBS pH 7.4 in glass tubes. Then, protein was precipitated at 95 °C for 30 min and the tubes were centrifuged (5 min at 10,000*g*). The clear supernatant was ultrafiltered (30 kDa filter-units, VWR centrifugal filter, VWR International, Darmstadt, Germany) to remove remaining larger proteins and the free IS concentration was determined in the resulting filtrate. The obtained clear filtrates were subjected to RP-HPLC (Gynkothek pump M480, auto-sampler Gynkothek Gina 50, on-line degasser ERC-3315a and column oven Gynkothek STH; Gynkothek/Dionex, Idstein, Germany). The IS concentration was measured using a C18 column (ProntoSIL Hypersorb ODS 3.0 µm, 250 × 4.6mm, Bischoff Chromatography, Leonberg, Germany) and fluorescence detection (spectrofluorometric detector RF-551, Shimadzu, Kyoto, Japan; λ_e__x_ = 280 nm/λ_em_ = 340 nm). The samples were eluted at 30 °C using a gradient from 15% to 25% *v*/*v* of solvent A (50mM NH_4_COOH buffer (HPLC grade, Fluka, Sigma Aldrich, St. Louis, MO, USA) pH 3.4) and solvent B (acetonitrile (LiChrosolv^®^, Merck, New York, NY, USA)) at a flow-rate of 1 mL/min.

### 4.7. Determination of the Bound IS Fraction

The protein bound fraction was calculated as follows:

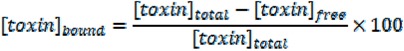



The ratio *K*_D_/*B*_m_ was calculated from the respective values of *K*_D_ and *B*_m_ obtained from the experimental free and bound IS concentrations. The ratio *K*_D_/*B*_m_ served for the calculation of the theoretical protein bound fraction, using Equation (1). Theoretical and experimental protein bound fractions were compared at low toxin-albumin ratio (α = 0.1). The protein bound fractions were determined as follows: for the theoretical approach, α was set at 0.1; for the experimental approach, α was obtained from the total toxin concentration measured by HPLC and the albumin concentration determined using laser nephelometry (BN ProSpec, Siemens, Germany):

*a_experimental_* = [*IS*]/[*HSA*]

### 4.8. Data Analysis

If not differently indicated, results are presented as mean ± SD of three different donors (*n* = 3). The comparative analysis of the binding affinity and capacity in human plasma was performed by using the paired Student’s *t*-test. Differences between uremic *versus* healthy plasma were analyzed by applying the Mann-Whitney-*U*-test, if the results were not normally distributed, and with a one-way ANOVA followed by the Tukey’s *post hoc* test, if normally distributed. A *p*-value < 0.05 was considered significant.

## 5. Conclusions

Increasing the ionic strength by modification of the NaCl concentration as well as higher temperature are effective to reduce the protein binding affinity and, consequently, the protein bound fraction of the uremic toxin IS in human plasma. Dilution further decreases the protein bound fraction by lowering the binding capacity of the plasma proteins. As only the free toxin fraction is removed during hemodialysis, these measures represent interesting approaches to enhance the removal of protein bound uremic toxins from blood, which may be beneficial for the outcome of patients undergoing dialysis.

## Abbreviations

*B*_m_: maximal binding capacity
ESRD: end-stage renal disease
IS: indoxyl sulfate
*K*_A_: association constant
*K*_D_: dissociation constant
NaCl: sodium chloride
RP-HPLC: reversed-phase high performance liquid chromatography

## Supplementary Files

Supplementary File 1 Supplementary Information (PDF, 230 KB)
