# Supplementary material for: Binding Affinity and Capacity for the Uremic Toxin Indoxyl Sulfate
Source: Toxins (Basel). 2014 Jan 24;6(2):416–29. doi: 10.3390/toxins6020416 (PMC3942743; doi:10.3390/toxins6020416)
Supplement: Supplementary File 1 — Supplementary Information (PDF, 230 KB) [file toxins-06-00416-s001.pdf]

Supplemental Data Equation (1)

Protein bound toxins such as indoxyl sulfate (IS) may be described with the ligand-receptor theory since only non- covalent binding (for example via Van der Waals interactions) occurs. In this case the receptor is albumin, the main protein in plasma and also well-known to bind many hydrophobic compounds to its different binding sites, and the ligand is e.g., IS or any other uremic toxin. The free receptor R and free ligand L are always in equilibrium with the ligand-receptor complex RL (also: bound ligand to the receptor) according to the law of mass action:

$$R + L \leftrightarrow RL \tag{4}$$

Toxin binding to albumin depends on the dissociation constant K_D [M] and on the binding capacity B_m [M] which is proportional to the number of binding sites on the protein (hypothesis of one site specific binding), according to:

$$RL = \frac{B_m . L}{K_D + L} \tag{5}$$

It is possible to write $L_T = L + RL$, the total toxin concentration. Thus $L = L_T - RL$ and (Equation (5)) can be rewritten as follows:

$$RL = \frac{B_m . L_T - B_m . RL}{K_D + L_T - RL} \tag{6}$$

This leads to:

$$RL. (K_D + L_T + B_m) - RL^2 - B_m . L_T = 0 \tag{7}$$

To solve Equation (7), only one physical possible solution is admitted given as:

$$RL = \frac{K_D + B_m + L_T - \sqrt{(K_D + B_m + L_T)^2 - 4. B_m . L_T}}{2} \tag{8}$$

Since the protein bound fraction PBF is given by

$$PBF = \frac{RL}{L_T}$$

We obtain,

$$PBF = \frac{K_D + B_m + L_T - \sqrt{(K_D + B_m + L_T)^2 - 4. B_m . L_T}}{2. L_T} \tag{9}$$

Equation (9) may be simplified by introducing α as the toxin/receptor ratio with $\alpha = L_T/B_m$ in order to write the PBF as a function of the total toxin concentration and toxin properties:

$$PBF = \frac{\frac{K_D}{B_m} + \alpha + 1 - \sqrt{\left(\frac{K_D}{B_m} + \alpha + 1\right)^2 - 4. \alpha}}{2. \alpha} \tag{1}$$

© 2014 by the authors; licensee MDPI, Basel, Switzerland. This article is an open access article distributed under the terms and conditions of the Creative Commons Attribution license (http://creativecommons.org/licenses/by/3.0/).
